# Supplementary material for: A computational spatial whole-Cell model for hepatitis B viral infection and drug interactions
Source: Sci Rep. 2023 Dec 4;13:21392. doi: 10.1038/s41598-023-45998-0 (PMC10695947; doi:10.1038/s41598-023-45998-0)
Supplement: Supplementary file 1 — Supplementary Information. [file 41598_2023_45998_MOESM1_ESM.pdf]

# Supplementary Information
# A Computational Spatial Whole-Cell Model for Hepatitis B Viral Infection and Drug Interactions

Zhaleh Ghaemi,[1,2]* Oluwadara Nafiu,[3] Emad Tajkhorshid,[1,2,4,5] Martin Gruebele,[1,2,3,5,6] and Jianming Hu[7,8]

*[1]Department of Chemistry, University of Illinois at Urbana-Champaign, Urbana, IL 61801, USA*
*[2]Beckman Institute for Advanced Science and Technology, University of Illinois at Urbana-Champaign, Urbana, IL 61801, USA*
*[3]Carle-Illinois College of Medicine, University of Illinois at Urbana-Champaign, Urbana, IL 61801, USA*
*[4]Department of Biochemistry, University of Illinois at Urbana-Champaign, Urbana, IL 61801, USA*
*[5]Center for Biophysics and Quantitative Biology, University of Illinois at Urbana-Champaign, Urbana, IL 61801, USA*
*[6]Department of Physics, University of Illinois at Urbana-Champaign, Urbana, IL 61801, USA*
*[7]Department of Microbiology and Immunology, Pennsylvania State University, Hershey, PA 17033, USA*

*Corresponding author: ghaemi@illinois.edu

Table S1. Diffusion coefficients of species involved in the reactions

| Species | Diffusion coeff. | Compartment | Reference |
|---|---|---|---|
| $capsid_{DNA}$ | $7.4\times10^{-12}$ | Cytoplasm/ER | [1] |
| $capsid_{DNA}$ | $16\times10^{-12}$ | NPC | [1] |
| $capsid_{empty}$ | $7.4\times10^{-12}$ | Cytoplasm/ER | [1] |
| $capsid_{empty}$ | $16\times10^{-12}$ | NPC | [1] |
| $capsid_{RNA}$ | $7.4\times10^{-12}$ | Cytoplasm/ER | [1] |
| $capsid_{RNA}$ | $16\times10^{-12}$ | NPC | [1] |
| $viral_{pol}$ | $22\times10^{-12}$ | Cytoplasm | Equals to L (assumption) |
| $RNA_{pol}$ | $22\times10^{-12}$ | Nucleus | Equals to $viral_{pol}$ (assumption) |
| $cccDNA.RNA_{pol}$ | $22\times10^{-12}$ | Nucleus | Equals to $viral_{pol}$ (assumption) |
| rcDNA | $0.07\times10^{-12}$ | NPC | [2] |
| rcDNA | $0.61\times10^{-12}$ | Nucleus | [2] |
| cccDNA | $0.61\times10^{-12}$ | Nucleus | [2] |
| LSmRNA | $0.61\times10^{-12}$ | Nucleus | [2] |
| pgRNA | $0.61\times10^{-12}$ | Nucleus | [2] |
| SmRNA | $0.61\times10^{-12}$ | Nucleus | [2] |
| XmRNA | $0.61\times s10^{-12}$ | Nucleus | [2] |

| | | | |
|---|---|---|---|
| preCmRNA | $0.61\times10^{-12}$ | Nucleus | [2] |
| LSmRNA | $2.24\times10^{-12}$ | Cytoplasm | [2] |
| pgRNA | $2.24\times10^{-12}$ | Cytoplasm | [2] |
| SmRNA | $2.24\times10^{-12}$ | Cytoplasm | [2] |
| XmRNA | $2.24\times10^{-12}$ | Cytoplasm | [2] |
| preCmRNA | $2.24\times10^{-12}$ | Cytoplasm | [2] |
| LSmRNA | $0.07\times10^{-12}$ | NPC | [2] |
| pgRNA | $0.07\times10^{-12}$ | NPC | [2] |
| SmRNA | $0.07\times10^{-12}$ | NPC | [2] |
| XmRNA | $0.07\times10^{-12}$ | NPC | [2] |
| preCmRNA | $0.07\times10^{-12}$ | NPC | [2] |
| preC | $22\times10^{-12}$ | Cytoplasm | Equals to L (assumption) |
| S | $22\times10^{-12}$ | Cytoplasm/ER | Equals to L (assumption) |
| M | $22\times10^{-12}$ | Cytoplasm/ER | Equals to L (assumption) |
| L | $22\times10^{-12}$ | Cytoplasm/ER | scaled diffusion coefficient of GFP[3] based on [4] |
| X | $22\times10^{-12}$ | Cytoplasm | Equals to L (assumption) |
| interm | $9.7\times10^{-12}$ | Cytoplasm | ref [1] scaled based on [4] |
| RNP | $2.24\times10^{-12}$ | Cytoplasm | Equals to RNAs in cytoplasm |
| capsid$_{DNA}$.L(1,…20) | $22\times10^{-12}$ | ER | Equals to L (assumption) |
| capsid$_{RNA}$.L(1,…20) | $22\times10^{-12}$ | ER | Equals to L (assumption) |
| capsid$_{empty}$.L(1,…20) | $22\times10^{-12}$ | ER | Equals to L (assumption) |

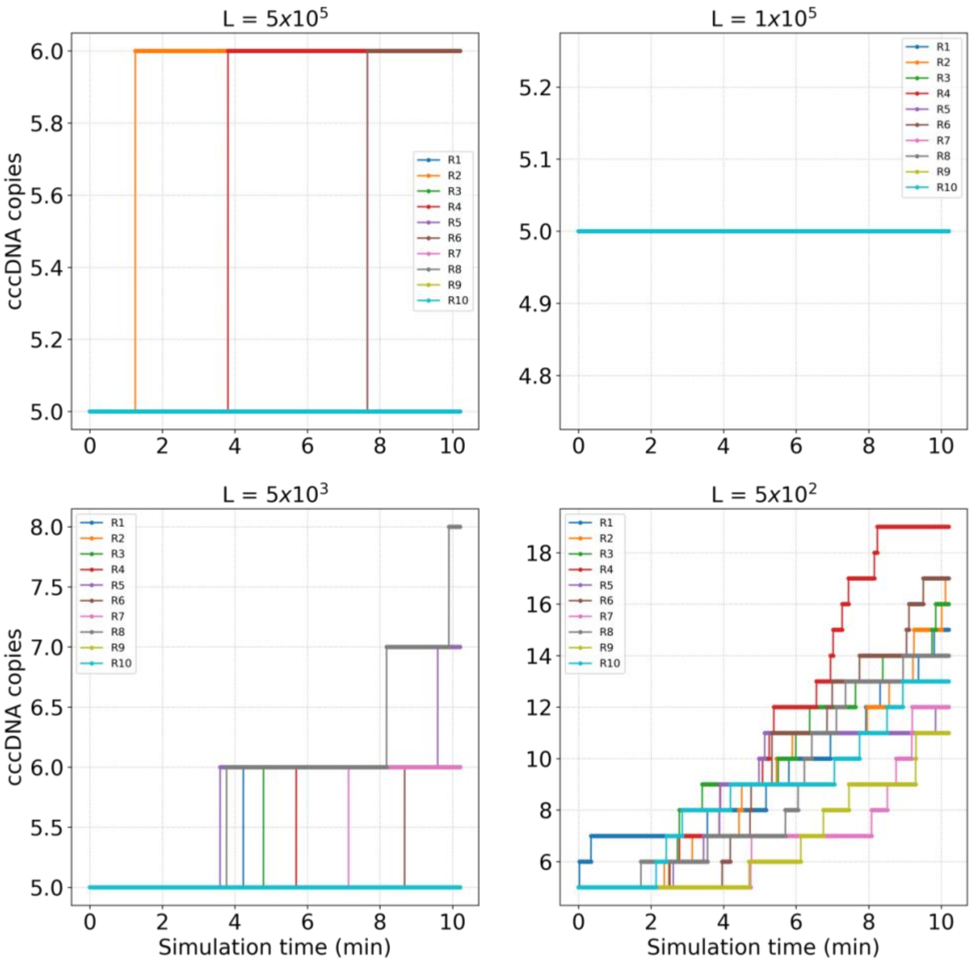


Figure S1. Trajectory of cccDNA copy numbers as a function of simulation time for various L protein abundance for all simulation replicas (R1 to R10). As the copy number of L proteins reduces the dominant pathway switches from secretion to amplification and the cccDNA copy numbers increases. Therefore, for L=5x102 the cccDNA copy number increases such that it reaches an average value of ~ 15 within 10 minutes of simulation time.

Figure S2 shows how the 3D distribution and counts of capsids and cccDNA species change during the simulation time: At the start of a simulation (Figure S2-A), capsids are randomly distributed in the cytoplasm, with 5 cccDNA already present in the nucleus; At the end of the 10 mins of simulation (Figure S2-B), the progress of the infection is shown by an increase in the number of cccDNA. The majority of the mature and empty capsids are bound to the NPCs to be disassembled.

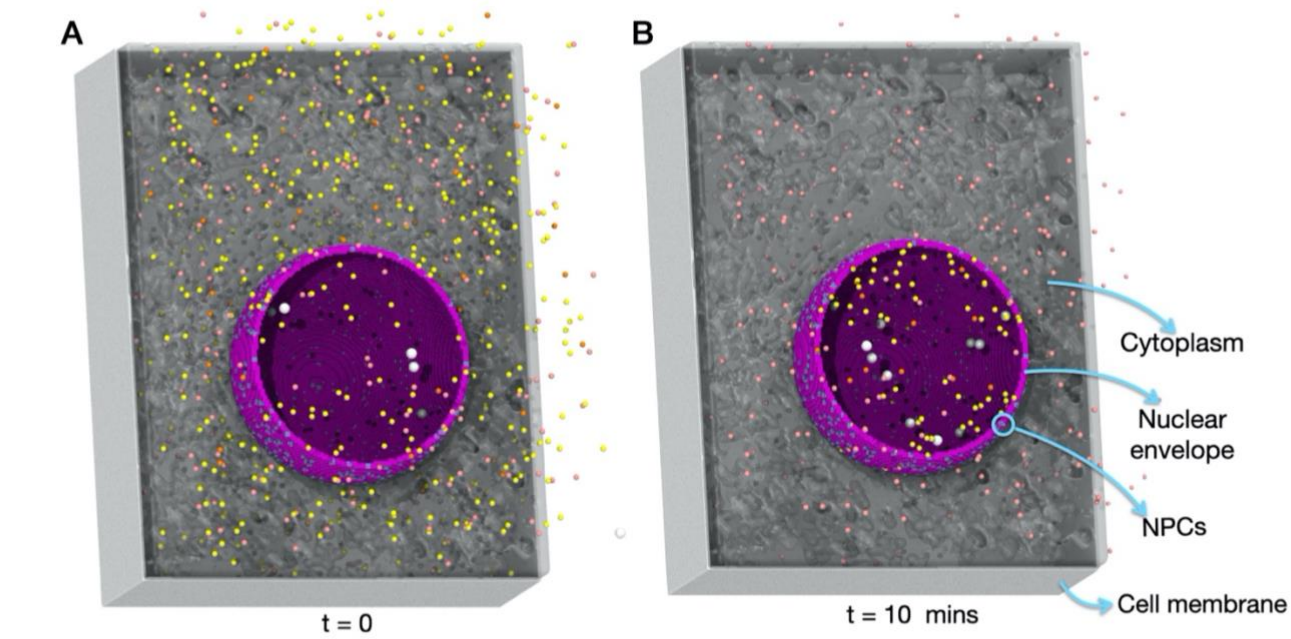


Figure S2: (A) the empty (yellow), RNA-containing (pink) and mature (orange) capsids are randomly distributed in the cytoplasm (shaded grey region), with about 5 cccDNA (white) already present in the nucleus. (B) After 10 mins of simulation, the number of cccDNA increases and some of the capsids are bound to the NPCs before being disassembled.

Table S2. Reactions describing the degradation of different species that were included in the kinetic model

| Degradation reactions | Rate | Unit | Compartment |
|---|---|---|---|
| rcDNA → 0 | $1.60\times10^{-7}$ | $s^{-1}$ | Nucleus |
| cccDNA→ 0 | $1.60\times10^{-7}$ | $s^{-1}$ | Nucleus |
| pgRNA→ 0 | $3.8\times10^{-5}$ | $s^{-1}$ | Cytoplasm |
| LSmRNA→ 0 | $6.4\times10^{-5}$ | $s^{-1}$ | Cytoplasm |
| SmRNA→ 0 | $6.4\times10^{-5}$ | $s^{-1}$ | Cytoplasm |
| XmRNA→ 0 | $1.9\times10^{-4}$ | $s^{-1}$ | Cytoplasm |
| preCmRNA→ 0 | $3.8\times10^{-5}$ | $s^{-1}$ | Cytoplasm |
| L→ 0 | $2.9\times10^{-4}$ | $s^{-1}$ | Cytoplasm |
| M→ 0 | $2.9\times10^{-4}$ | $s^{-1}$ | Cytoplasm |
| S→ 0 | $2.9\times10^{-4}$ | $s^{-1}$ | Cytoplasm |
| dimer→ 0 | $2.9\times10^{-4}$ | $s^{-1}$ | Cytoplasm |
| $viral_{pol}$→ 0 | $2.9\times10^{-4}$ | $s^{-1}$ | Cytoplasm |

## References


(1) Lill, Y.; Lill, M. A.; Fahrenkrog, B.; Schwarz-Herion, K.; Paulillo, S.; Aebi, U.; Hecht, B. Single Hepatitis-B Virus Core Capsid Binding to Individual Nuclear Pore Complexes in Hela Cells. *Biophys. J.* **2006**, *91* (8), 3123–3130. https://doi.org/10.1529/biophysj.106.087650.

(2) Ma, J.; Liu, Z.; Michelotti, N.; Pitchiaya, S.; Veerapaneni, R.; Androsavich, J. R.; Walter, N. G.; Yang, W. High-Resolution Three-Dimensional Mapping of MRNA Export through the Nuclear Pore. *Nat. Commun.* **2013**, *4* (2414).

(3) Mika, J. T.; Schavemaker, P. E.; Krasnikov, V.; Poolman, B. Impact of Osmotic Stress on Protein Diffusion in Lactococcus Lactis. *Mol. Microbiol.* **2014**, *94* (4), 857–870. https://doi.org/https://doi.org/10.1111/mmi.12800.

(4) Dill, K. a; Ghosh, K.; Schmit, J. D. Physical Limits of Cells and Proteomes. *Proc. Natl. Acad. Sci. U. S. A.* **2011**, *108* (44), 17876–17882. https://doi.org/10.1073/pnas.1114477108.
